# Drosophila Sperm Swim Backwards in the Female Reproductive Tract and Are Activated via TRPP2 Ion Channels

**DOI:** 10.1371/journal.pone.0020031

**Published:** 2011-05-20

**Authors:** Michael Köttgen, Alexis Hofherr, Weizhe Li, Kristy Chu, Stacey Cook, Craig Montell, Terry Watnick

**Affiliations:** 1 Division of Nephrology, Department of Medicine, Johns Hopkins University School of Medicine, Baltimore, Maryland, United States of America; 2 Departments of Biological Chemistry and Neuroscience, Center for Sensory Biology, Johns Hopkins University School of Medicine, Baltimore, Maryland, United States of America; Yale School of Medicine, United States of America

## Abstract

**Background:**

Sperm have but one purpose, to fertilize an egg. In various species including *Drosophila melanogaster* female sperm storage is a necessary step in the reproductive process. Amo is a homolog of the human transient receptor potential channel TRPP2 (also known as PKD2), which is mutated in autosomal dominant polycystic kidney disease. In flies Amo is required for sperm storage. *Drosophila* males with Amo mutations produce motile sperm that are transferred to the uterus but they do not reach the female storage organs. Therefore Amo appears to be a mediator of directed sperm motility in the female reproductive tract but the underlying mechanism is unknown.

**Methodology/Principal Findings:**

Amo exhibits a unique expression pattern during spermatogenesis. In spermatocytes, Amo is restricted to the endoplasmic reticulum (ER) whereas in mature sperm, Amo clusters at the distal tip of the sperm tail. Here we show that flagellar localization of Amo is required for sperm storage. This raised the question of how Amo at the rear end of sperm regulates forward movement into the storage organs. In order to address this question, we used *in vivo* imaging of dual labelled sperm to demonstrate that *Drosophila* sperm navigate backwards in the female reproductive tract. In addition, we show that sperm exhibit hyperactivation upon transfer to the uterus. A*mo* mutant sperm remain capable of reverse motility but fail to display hyperactivation and directed movement, suggesting that these functions are required for sperm storage in flies.

**Conclusions/Significance:**

Amo is part of a signalling complex at the leading edge of the sperm tail that modulates flagellar beating and that guides a backwards path into the storage organs. Our data support an evolutionarily conserved role for TRPP2 channels in cilia.

## Introduction

The evolutionary success of sexual reproduction depends on the ability of motile sperm cells to find an egg and fertilize it. Different animals have evolved distinct and elaborate mechanisms to accomplish this essential task but female sperm storage is a commonly used strategy in species ranging from insects to mammals. In *Drosophila melanogaster*, female sperm storage is critical for maximal reproductive success [1]. Approximately 4000 sperm are transferred to a *Drosophila* female during mating but ∼80% are expelled from the uterus when the first egg is laid [2]. The remaining sperm are stored for up to two weeks in two types of female storage organs, a single seminal receptacle and a pair of spermathecae [2]. Stored sperm can be used for fertilization in the absence of continued mating.

There is relatively little known about the factors that govern sperm storage in the female reproductive tract. In flies, male accessory gland proteins contribute to this process and sperm derived from *Drosophila* males that are lacking the seminal fluid protein Acp36DE are inefficiently transferred from the uterus to the sperm storage organs [3], [4], [5]. Acp36DE is thought to mediate sperm storage by inducing favourable conformational changes in the female reproductive tract [5].

We have previously shown that Amo is a sperm enriched protein that is essential for fertility and sperm storage in *Drosophila melanogaster* [6]. Amo is a homolog of the human transient receptor potential channel TRPP2, encoded by the Polycystic Kidney Disease 2 gene (*PKD2*) [6], [7]. Mutations in human *PKD2* result in autosomal dominant polycystic kidney disease [8]. TRPP2 channels are evolutionarily conserved, calcium-permeable nonselective cation channels that are localized in the endoplasmic reticulum (ER) and in cilia, but the physiological function of these ion channels *in vivo* is poorly understood [9], [10], [11], [12], [13], [14]. *Drosophila* males with *amo* mutations are sterile; they produce motile sperm that are transferred to the uterus but the sperm do not reach the female storage organs [6], [7]. Taken together the data suggests that Amo could be a mediator of directional sperm movement.

In the present study we investigate the role of Amo in sperm motility within the female reproductive tract. We show that although Amo is expressed in the endoplasmic reticulum (ER) during earlier stages of spermatogenesis, it is the flagellar localization at the tip of the sperm tail, which is critical for making sperm storage competent. This finding coupled with Amo's role in directional sperm movement prompted us to hypothesize that *Drosophila* sperm might swim tail first. We used *in vivo* imaging of dual labelled sperm to demonstrate that wild type *Drosophila* sperm do in fact navigate backwards in the female reproductive tract. In addition, we discovered that sperm exhibit activated flagellar beating upon transfer to the uterus. Amo mutant sperm remain capable of reverse motility but fail to display hyperactivation, suggesting that activated flagellar beating is a requirement for sperm storage in flies.

## Results

### Flagellar Amo is required for proper Sperm Storage

**Figure 1:**
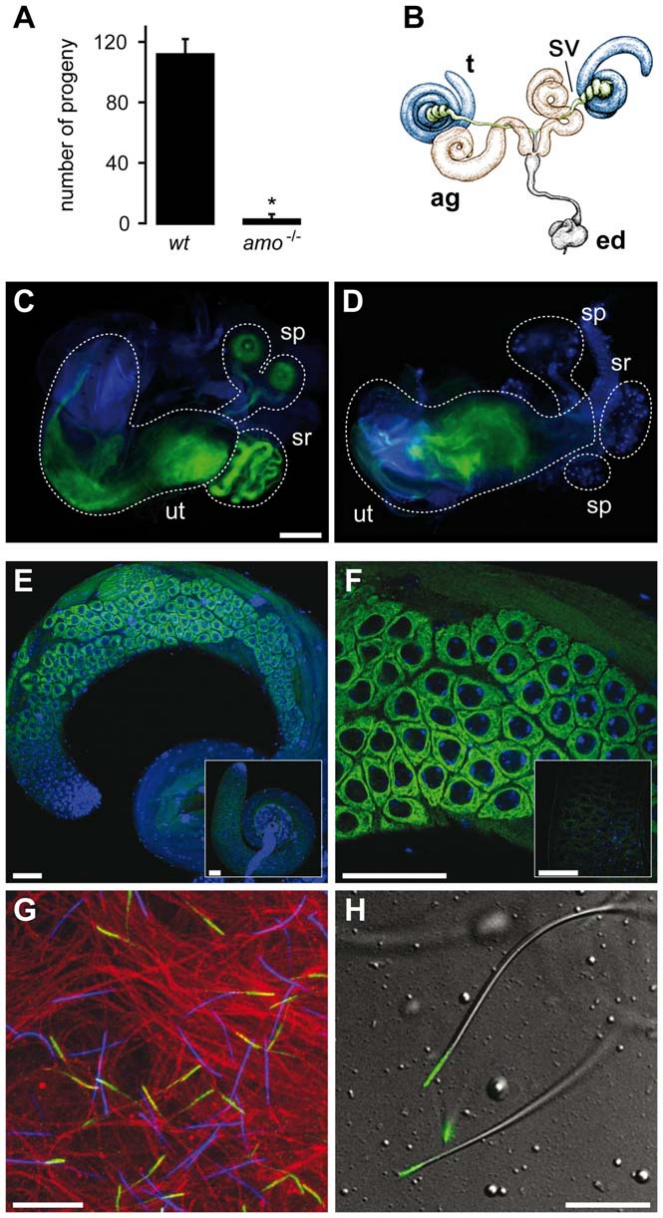
Amo is Expressed in the Male Germ Line and is Required for Sperm Storage in *Drosophila*. **A.** *Amo* mutant males are sterile. Progeny produced from mating wild type and *amo^−/−^* males with wild type females (n = 10). **B.** Schematic model of the *Drosophila* male reproductive system. Testis (t), seminal vesicles (sv), accessory gland (ag), ejaculatory duct (ed). **C,** **D.** Female reproductive tract after mating with wild type (**C**) or *amo^−/−^* sperm (**D**) (sperm tails labelled with dj-GFP: green, DAPI-labelled nuclei: blue; scale bar: 100 µm). In (**D**) there are no sperm in either the seminal receptacle or the spermathecae. **E.** Expression pattern of Amo in testis (anti-Amo: green, nuclear DAPI staining: blue; scale bars 50 µm); inset: Amo staining in *amo^−/−^* testis. **F.** Intracellular localization of Amo in spermatocytes (scale bars 50 µm); inset: Amo staining in *amo^−/−^* testis. **G.** Fluorescence labelling of mature sperm (anti-Amo: green, concavalin A: red, DAPI: blue; scale bar 20 µm). **H.** Localization of Amo in mature sperm (anti-Amo: green; scale bar 20 µm).

*Drosophila* males with Amo mutations produce motile sperm that are transferred to the uterus but do not reach the female storage organs (Figure 1A–D, Figure S1A–C and Video S1 and S2). To gain insights into the physiological function of Amo we studied its cellular distribution in the male germline. Amo exhibits a unique and distinct expression pattern during spermatogenesis (Figure 1). In spermatocytes, Amo is localized in the endoplasmic reticulum (ER, Figure 1 E and F) as shown by co-localization with the ER marker protein disulfide isomerase (Figure S2) [15]. In mature sperm, however, Amo clusters at the tip of the sperm tail (Figure 1G and H). This expression pattern closely resembles the distribution of mammalian TRPP2, which is also found in the ER and in primary cilia [10]. It is unknown which localization of TRPP2 is functionally important. In *Drosophila*, Amo might be required in the ER of developing spermatocytes to make sperm storage-capable later in development. Alternatively, since TRPP2 channels are thought to have an evolutionarily conserved role in ciliary signalling, we hypothesized that the flagellar localization of this ion channel might be essential for regulating directional motility and sperm storage [16], [17].

**Figure 2:**
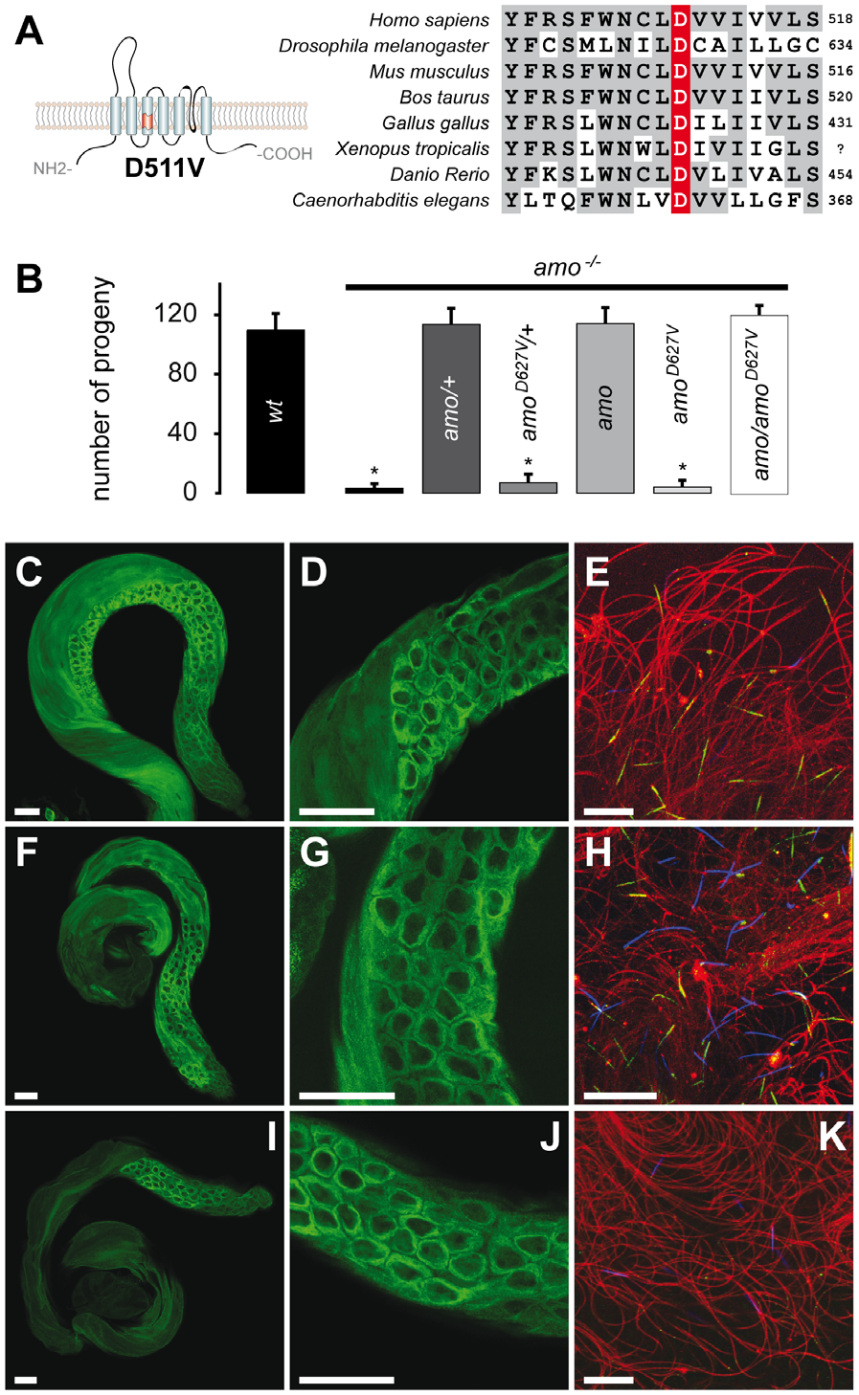
Amo Localization at the Tip of the Sperm Tail is Required for Sperm Storage. **A.** The human ADPKD patient mutation PKD2^D511V^ maps to the third transmembrane domain of PKD2, left panel. The aspartate in postition 511 (highlighted in red) is highly conserved throughout evolution (right panel). **B**. Fertility tests with *amo* mutant males mated to wild type females reveal that loss of *amo* is rescued by transgenic expression of either 1 or 2 copies wild type Amo but not by Amo^D627V^ on the 3^rd^ chromosome (black bars: no transgene, dark grey: one copy of transgenic Amo, light grey: two copies of the transgenes, white: transheterozygous males; N = 10). **C–K.** Subcellular localization of Amo in testis and mature sperm. Localization of native Amo in testis, spermatocytes and mature sperm of WT males (**C–E**), *amo* mutant males expressing a wild type Amo transgene (**F–H**) or the Amo^D627V^ transgene (**I–K**). Scale bars for testis and spermatocytes (C, D, F, G, I, J): 50 µm; for sperm tails (E, H, K): 20 µm.

In order to investigate this question, we took advantage of a mutation in human TRPP2, D511V, which causes autosomal dominant polycystic kidney disease [18]. This missense amino acid substitution has been reported to eliminate TRPP2 channel function *in vitro* and to act in a dominant negative fashion in over-expression systems [12], [19]. This is presumably because TRPP2 forms multimeric complexes that require all subunits to be functional. We mutated the corresponding aspartate in *Drosophila* Amo to valine (Amo^D627V^) (Figure 2A), and then expressed wild type and Amo^D627V^ transgenic channels in *amo* mutant flies. The wild type transgene rescued the sperm storage phenotype and its subcellular distribution was identical to that of the native protein (Figure 2B–H, Figure S1A–D, and Figure S3). In contrast, Amo^D627V^ was unable to restore normal levels of sperm storage (Figure 2B and Figure S1E). Although the mutant protein could be detected in the ER of spermatocytes (Figure 2I and J), it was absent at the tip of the mature sperm tail (Figure 2K), suggesting that this mutation resulted in a flagellar trafficking defect *in vivo*. To test whether Amo^D627V^ acts in a dominant negative fashion we co-expressed wild type and mutant Amo^D627V^ in *amo* null flies and found that male sterility could still be fully rescued (Figure 2B, Figure S1F, Figure S4, and Figure S6). Since both wild type and mutant forms of Amo are expressed in the ER but only wild type Amo is found at the tip of the sperm tail, the lack of a dominant negative effect is likely due to the failure of Amo^D627V^ to be incorporated into the flagellar pool of Amo, which is required for sperm storage. These results are consistent with the idea that localization at the distal end of the sperm tail is essential for Amo function *in vivo*.

### *Drosophila* Sperm Move Backwards in the female reproductive tract

**Figure 3:**
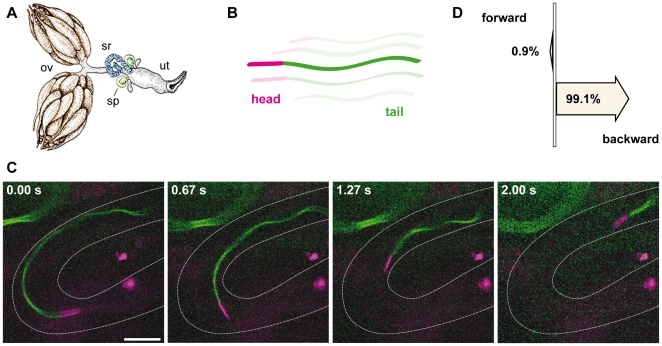
*Drosophila* Sperm Navigate Backwards in the Female Reproductive Tract. **A**. Schematic model of the *Drosophila* female reproductive system. Ovary (ov), spermathecae (sp), seminal receptacle (sr), uterus (ut). **B.** Schematic of a mature sperm, showing the acrosome in pink and the sperm tail in green. **C.** *Drosophila* sperm tracking in the seminal receptacle (sperm tail labeled with dj-GFP: green, sperm head labeled with Prot-B-DsRed: red). **D.** The vast majority of sperm swim backward (n = 222 sperm, N = 11 flies).

The requirement for Amo at the flagellar tip coupled with its pivotal role in directed sperm motility prompted us to ask how Amo could regulate head first movement? We hypothesized that sperm might travel in reverse or tail first rather than in a forward direction. In order to explore this possibility, we adapted methods that allowed us to assay directional sperm movement within the female reproductive tract in real time [20]. We generated male flies with dual colour sperm: sperm tails labelled with green fluorescent protein (GFP) and heads labelled with red fluorescent protein (RFP) (Figure 3). This combination of tags allowed us to use high-speed confocal microscopy to track the course and direction of sperm movement (Videos S1, S2, S3, S4, S5, and S6). We show that sperm in the female reproductive tract move backwards both in the uterus and in the seminal receptacle (Figure 3C and D and Video S3). Of 222 sperm observed in 11 independent experiments, all but two sperm heads trailed the sperm tail (Figure 3D). This establishes that *Drosophila* sperm swim backwards in the female reproductive tract *in vivo*. This pattern of directed motility has not been reported for sperm of any other species.

### *Amo* Mutant Sperm are Capable of Backward Swimming

The localization of Amo at the rear end of the flagellum makes it an appealing regulator of reverse sperm motility. One can envision two potential roles for Amo in this process. Amo might be required for the specific flagellar waveforms that result in backward motion. In this scenario, Amo mutant sperm would be expected to lack reverse motility. Alternatively, reverse motility could be the default direction for *Drosophila* sperm and Amo might act as a sensor at the leading edge, serving a distinct pathfinding function. In order to test these possibilities, we generated *amo* mutant flies producing dual labelled sperm and mated these males to wild type female. We found that *amo* mutant sperm still are capable of backwards swimming, both in the uterus and in the seminal receptacle (N = 13; of 143 sperm only 9 showed forward movement, Video S4). Therefore, factors other than impaired backward swimming directionality must cause the sperm storage defect in *amo* mutant sperm.

### *Drosophila* Sperm exhibit Amo-dependent Activation in the Uterus

**Figure 4:**
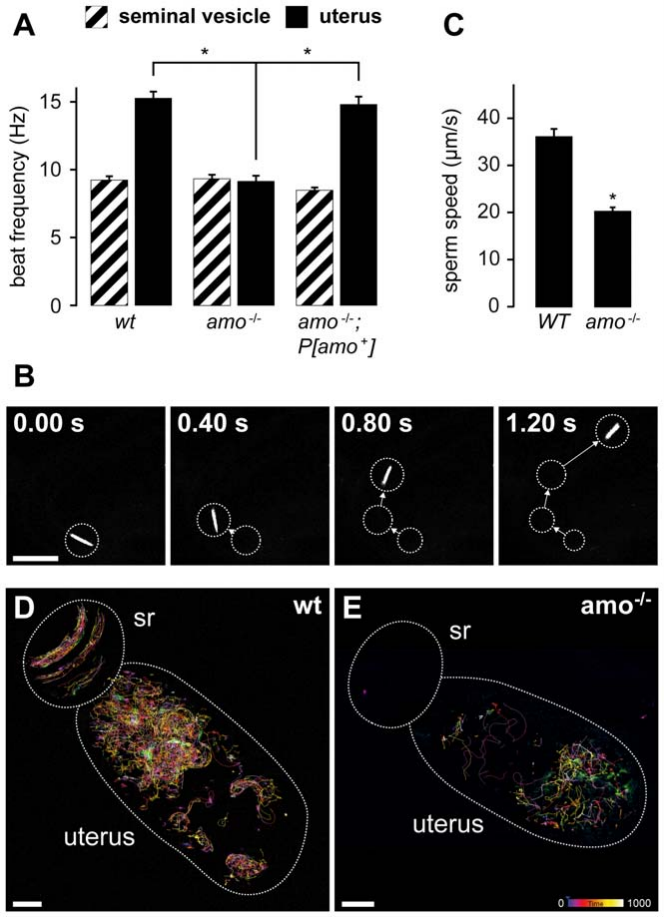
Amo Regulates Sperm Beat Frequency, Swimming Speed and Dynamic distribution in the Uterus. **A.** The sperm beat frequency is higher in sperm dissected from mated uteri (N = 22, wild type, solid bars) compared with sperm dissected from seminal vesicles (N = 26 wild type, hatched bars). The intrauterine increase in sperm beat frequency is absent in *amo* mutant sperm (N = 20 seminal vesicles, hatched bars N = 30 uteri solid bars) but is rescued upon expression of a wild type *amo* transgene in an *amo* null background (N = 22 seminal vesicles, N = 20 uteri). **B.** Tracking of an individual *amo^−/−^* sperm head (scale bar 20 µm). **C.** Average sperm speed in the female reproductive tract (calculated from N = 7 experiments). **D.** Representative tracking experiment of wild type sperm in the female reproductive tract (scale bar 40 µm, pseudocolor depicts the time of tracking during the experiment: 1000 frames/experiment). **E.** Representative tracking experiment of *amo* mutant sperm in the female reproductive tract (scale bar 20 µm).

To characterize sperm motility patterns in both wild type and *amo* mutant sperm we analyzed beat frequency and swimming speed *in vitro* and *in vivo,* respectively. We found that wild type sperm released from the uterus immediately after mating have a significantly higher beat frequency when compared to sperm released from the male seminal vesicle, suggesting that *Drosophila* sperm undergo an activation step similar to what has been described for capacitated mammalian sperm (Figure 4A) [21], [22]. Although the baseline beat frequency of *amo* mutant sperm was similar to wild type, they failed to demonstrate an increase in beat frequency when released from the uterus (Figure 4A). This defect in sperm activation was rescued by a wild type *amo* transgene. To test whether the decreased beat frequency of *amo* mutant sperm translated into altered swimming speed of sperm *in vivo*, we tracked the movement of sperm heads in the uterus (Figure 4B and Video S5). Consistent with the observed decrease in beat frequency, the swimming speed of *amo* mutant sperm in the uterus was reduced significantly when compared to wild type sperm (Figure 4C and Figure S5). In addition, the dynamic distribution of sperm was altered in the *amo* mutant. Immediately after mating, wild type sperm clustered near the entrance of the sperm storage organs in the upper third of the uterus (Figure 4D), whereas *amo* mutant sperm did not show this distribution pattern (Figure 4E). Taken together, these defects in sperm function are likely to explain the inability of *amo* mutant sperm to reach the female sperm storage organs.

## Discussion

In the current study we demonstrate that Amo, a member of the TRPP2 ion channel family, is required at the distal end of the sperm flagellum for directed sperm transit to the female storage organs. This is consistent with a conserved role for TRPP2 (aka polycystin-2 or PKD2) channels in ciliary structures. In *C. elegans*, TRPP2 is found at the ciliated endings of male specific sensory neurons where it is postulated to sense cues from its mate, resulting in stereotypical male mating behaviours [23], [24]. Similarly, in mammals, TRPP2 localizes to primary cilia on renal epithelial cells as well as embryonic node cells and is thought to function as a mechanosensitive channel [14], [25], [26], [27], [28]. Therefore, TRPP2 channels appear to play a sensory role in a variety of ciliary contexts.

Amo's unique distribution in the flagellum led us to investigate the behaviour of sperm in the female reproductive tract. Our studies reveal several novel findings. First, we used real-time analysis of sperm expressing red and green fluorescent proteins in the head and tail respectively to show that shortly after mating, wild type *Drosophila* sperm travel backwards in the uterus and seminal receptacle. As far as we know, this type of motility has not been reported for sperm of any other species. One potential rationale for this type of directional movement is that it would permit sperm to “back in” to the female storage organs. This is supported by analyses of sperm organization within the female seminal receptacle [29], [30]. Sperm in the proximal portion of the seminal receptacle have been observed to cluster in a dense mass with heads pointed toward the entrance of the receptacle while the tails extend distally. Teleologically this is an attractive arrangement since sperm clustered with their heads toward the opening of the seminal receptacle would be available to exit head first, facilitating fertilization of eggs arriving from the oviduct.

Secondly we show that wild type *Drosophila* sperm acquire hyperactivated flagellar beating upon transfer to the female uterus. This is similar to mammalian sperm capacitation, a process that “switches on” spermatozoa, thus rendering them capable of fertilizing an egg [22], [31]. Hyperactivated motility is a characteristic feature of the capacitated sperm phenotype and is critical for fertilization. Hyperactivation is required for penetration of the zona pellucida as well as for sperm release from the oviduct, which serves as a sperm storage reservoir in some mammals. Acquisition of hyperactivated motility appears to be triggered by an increase in intracellular calcium that depends on the activity of the Catsper family of sperm enriched calcium channels [32], [33].

Amo is a logical mediator of sperm motility in Drosophila. In addition to its favourable localization in the sperm tail, TRPP2 proteins are calcium permeable non-selective cation channels [17]. Analysis of sperm motility in Amo mutant sperm reveal that they are capable of generating a backward trajectory but they exhibit clear defects in hyperactivation and swimming speed and they fail to accumulate near the entrance to the storage organs. Therefore, reverse movement may be necessary but is not sufficient for sperm storage to occur.

In summary we show that Amo localization defines a unique niche at the leading edge of sperm, which are traveling tail first. Activation of Amo serves to modulate flagellar beating and guides a backward trajectory into the sperm storage organs. In keeping with an evolutionarily conserved sensory role for TRPP2 channels in cilia we postulate that Amo is ideally located to receive cues upon transfer to the female reproductive tract. The nature of the stimuli to which the TRPP2 channel complex responds remains a matter of investigation. But in the light of our data and recent evidence from vertebrate models it is tempting to speculate that ligands rather than mechanical cues are critical for triggering TRPP2-mediated signalling [16], [27], [34].

## Materials and Methods

### Flies and husbandry

*Amo* knockout flies (*amo^1^*) have been described [6]. Transgenic flies expressing wild type and mutant Amo (D627V) were generated by BestGene Inc. (USA) using site-specific recombination with an attP landing site on the second chromosome (Bloomington stock number 9732) [35]. The genomic rescue construct contained the genomic region of *amo,* (CG6504) and approximately 1 kb of 5′ flanking sequence [6]. The mutation in the *amo* (CG6504) genomic rescue construct was generated by site-directed mutatgenesis using standard procedures. We sequenced all constructs to verify that no errors were introduced by PCR. Flies expressing the wild type and mutant genomic rescue constructs were crossed into the *amo* null background for analysis of fertility, sperm storage and sperm dynamics. Flies expressing Protamine-B-labelled with red fluorescent protein (ProtB-DsRed) on the third chromosome were kindly provided by John Belote and a line expressing don-juan-GFP (dj-GFP) on the third chromosome was obtained from Barbara Wakimoto [20], [36]. These lines were recombined to yield a strain expressing both fluorescent proteins on the same third chromosome. Flies expressing GFP tagged protein disulfide isomerase were obtained from Bloomington (stock number 6839). All flies were reared according to standard procedures and maintained at 25°C.

### Immunofluorescence

Dissection and preparation of testis and sperm as well as the anti-Amo antiserum (1∶3000) have been described [6]. Anti-rabbit Alexa fluor**®** 488 antibodies (1∶1000; Molecular Probes, USA) were used for visualization. Sperm tails were stained with Alexa fluor**®**-594 conjugated concavalin A (dilution 1∶20, Molecular Probes, USA) and sperm heads by 4′,6-Diamidin-2-phenylindol (DAPI). Images were recorded using a Zeiss LSM510 confocal microscope (Zeiss, Germany).

### Fertility assay

Males of various genotypes were separated upon eclosion and maintained in isolation 3 days prior to mating. Single pair matings with *w^1118^* (wt) virgin females were performed for 5 days. At that time both parents were removed from the vial. The number of progeny that eclosed from each vial was counted. Ten vials were scored for each genotype.

### Analysis of sperm beat frequency

Three-day old virgin male and/or female flies were used for these studies. Males were mated to *w^1118^* females and mating was interrupted after 20 minutes. To analyze the beat frequency, sperm were released from a seminal vesicle or from a mated-uterus into a Petri dish containing HEPES-buffered saline solution (145 mM NaCl, 4 mM KCl, 1 mM MgCl_2_, 1.3 mM CaCl_2_, 5 mM D-glucose, 10 mM 4-(2-hydroxyethyl)-1-piperazineethanesulfonic acid (HEPES), pH 7.4). Seven areas around the sperm mass were recorded. Three sperm tails per area were analyzed. One hundred fifty frames were acquired per area at a frame rate of 50 Hz. Sperm beating was recorded on an inverted microscope (DIC 20x, Olympus IX81, USA) with a charge-coupled camera device (Hamamatsu C9100-02; Hamamatsu Photonics, USA). Beat frequency was analysed using ImageJ software (NIH, USA, http://rsb.info.nih.gov/ij/).

### Live imaging of sperm in the female reproductive tract

Freshly hatched males (dj-GFP, ProtB-DsRed in a wild type or *amo^−/−^* background) and virgin *w^1118^* females were separated for four days prior to mating. Immediately after termination of mating, female flies were anaesthetised with CO_2_ and dissected in HEPES-buffered saline solution using fine forceps. The lower reproductive tract was removed without compression of the uterus by gently severing the ovipositor from surrounding cuticle and longitudinally opening the mid-ventrum. The digestive tract was severed close to the anus and the ovaries were removed at the base of the lateral oviducts. Female reproductive tracts were aspirated using a Pasteur pipette and transferred to a 15 µm slide 2×9 well (ibidi GmbH, Martinsried, Germany, #81806), containing 70 µl HEPES-buffered saline solution per well. Real-time imaging of dual-colour fluorescently labelled sperm in the female reproductive tract was performed on a microscope ZEISS LSM 510 DUO equipped with a LD LCI Plan-Apochromat 25x/0.8 glycerine objective (both from Carl Zeiss MicroImaging, Jena, Germany). Microscopic analysis was started within five minutes after unsolicited termination of mating. Excitation of the fluorophores (GFP and DsRed) was performed at 489 and 532 nm, respectively. For simultaneous detection of the red and green fluorescence, the LSM 5 Live confocal scanner was used, collecting emitted fluorescence in the range 495–525 nm for the dj-GFP fusion protein, and 560–675 nm for ProtB-DsRed. 5000 images were recorded per sample at 15 frames per second with 512×512 pixels and a pixel dwell time of 112 µs. (acquisition software: ZEN 2009, Carl Zeiss MicroImaging, Jena, Germany). Image analysis and sperm tracking were performed using Imaris tracking software (Bitplane, Zurich, Switzerland).

### Statistics

Data are presented as mean values ± s.e.m. (N = number of experiments, n = observations within an experiment). Unpaired student's t-Test was used for statistical analysis between two groups. Analysis of sperm dynamics and subcellular localization of TRPP2 wild type and mutant transgenes was performed in a blinded fashion.

## Supporting Information

Figure S1 **Sperm Storage Organs Dissected From Females Mated With Wild Type or** ***amo^−/−^*** **Flies.** A. Schematic model of the female reproductive system. Ovary (ov), spermathecae (sp), seminal receptacle (sr), uterus (ut). B**–**F. Seminal receptacles dissected 30**–**60 minutes after observed mating. Wild type virgin females were mated to males of different genotypes as indicated: **B.** Wild type, **C.** *amo* *^−/−^*, **D**. *amo* *^−/−^* *;P[amo],* **E.** *amo* *^−/−^* *;P[amo^D627V^],* and **F.** *amo* *^−/−^* *;P[amo/amo^D627V^]*.
(TIF)

Figure S2 **Amo Localizes to the Endoplasmic Reticulum (ER) in Spermatocytes.** A. Intracellular localization of Amo in spermatocytes (Anti-Amo 1∶3000, scale bar 20 µm). B. Expression pattern of the ER marker PDI-GFP in spermatocytes. **C.** Merged images.
(TIF)

Figure S3 **Amo Expression by Western Blot Analysis.** Lysates were prepared from male flies of various genotypes and subjected to immunoprecipitation with anti-Amo antisera. Western blots were probed with anti-Amo antisera.
(TIF)

Figure S4 **Subcellular Localization of Amo in Amo/Amo^D627V^ Transheterozygous Sperm.** Immunofluorescent labeling of sperm of the genotype *amo* *^−/−^* *; P[amo/amo^D627V^]*. **A**. DAPI. **B.** Concavalin A. **C.** Anti-Amo. **D.** Merged image. Scale bar 20 µm.
(TIF)

Figure S5 **Analysis of Sperm Speed in the Uterus.** Frequency distribution of sperm speed in the female reproductive tract (wt: green, *amo* ***^−/−^***: blue, N = 7 for each genotype).
(TIF)

Figure S6 **Absence of a Dominant Negative Effect of Amo^D627V^.** Fertility tests using heterozygous *amo^+/^* ^***−***^ mutant males show that introduction of one (dark grey bars) or two copies (light grey bars) of transgenic Amo^D627V^ (3^rd^ chromosome) does not result in impaired fertility.
(TIF)

Video S1 **Wild Type Sperm in the Female reproductive Tract.** Live imaging of wild type sperm bearing dj-GFP and ProtB-DsRed transgenes in the female uterus and seminal receptacle. Green fluorescent protein (sperm tails, green) and red fluorescent protein (sperm heads, magenta) were detected simultaneously. Isolated sperm head movement (white) in the uterus and the seminal receptacle is also demonstrated.
(MOV)

Video S2 ***Amo^−/−^*** **in the Female Reproductive Tract.** Live imaging of *amo^−/−^* sperm bearing dj-GFP and ProtB-DsRed transgenes in the female reproductive tract. Since *amo* *^−/−^* sperm do not reach the storage organs, there are no sperm visualized in the seminal receptacle. There is only background autofluorescence detected. Isolated Sperm head movement (white) in the uterus is also demonstrated.
(MOV)

Video S3 **Single Sperm Movement in the Female Reproductive Tract.** Live imaging of a single wild type sperm labelled with dj-GFP and ProtB-DsRed transgenes in the seminal receptacle is demonstrated. The sperm moves tail first.
(MOV)

Video S4 **Single Sperm Movement of** ***amo*** ***^−/−^*** **Sperm in the Female Reproductive Tract.** Live imaging of a single *amo* *^−/−^* sperm labelled with dj-GFP and ProtB-DsRed transgenes. The sperm moves backwards.
(MOV)

Video S5 **Tracking of Sperm in the Female Reproductive Tract.** Representative tracking of sperm heads in the uterus. Each sperm head (magenta) is marked with a dot. The pseudocolor bar at the right represents the time of tracking during the experiment.
(MOV)

Video S6 **Wild Type Sperm in the Female Reproductive Tract.** Real-time imaging of dual-colour fluorescently labelled wild type sperm in the seminal receptacle and uterus.
(MOV)
