# Supplementary figures and images for: Drosophila Sperm Swim Backwards in the Female Reproductive Tract and Are Activated via TRPP2 Ion Channels
Source: PLoS One. 2011 May 20;6(5):e20031. doi: 10.1371/journal.pone.0020031 (PMC3098850; doi:10.1371/journal.pone.0020031)

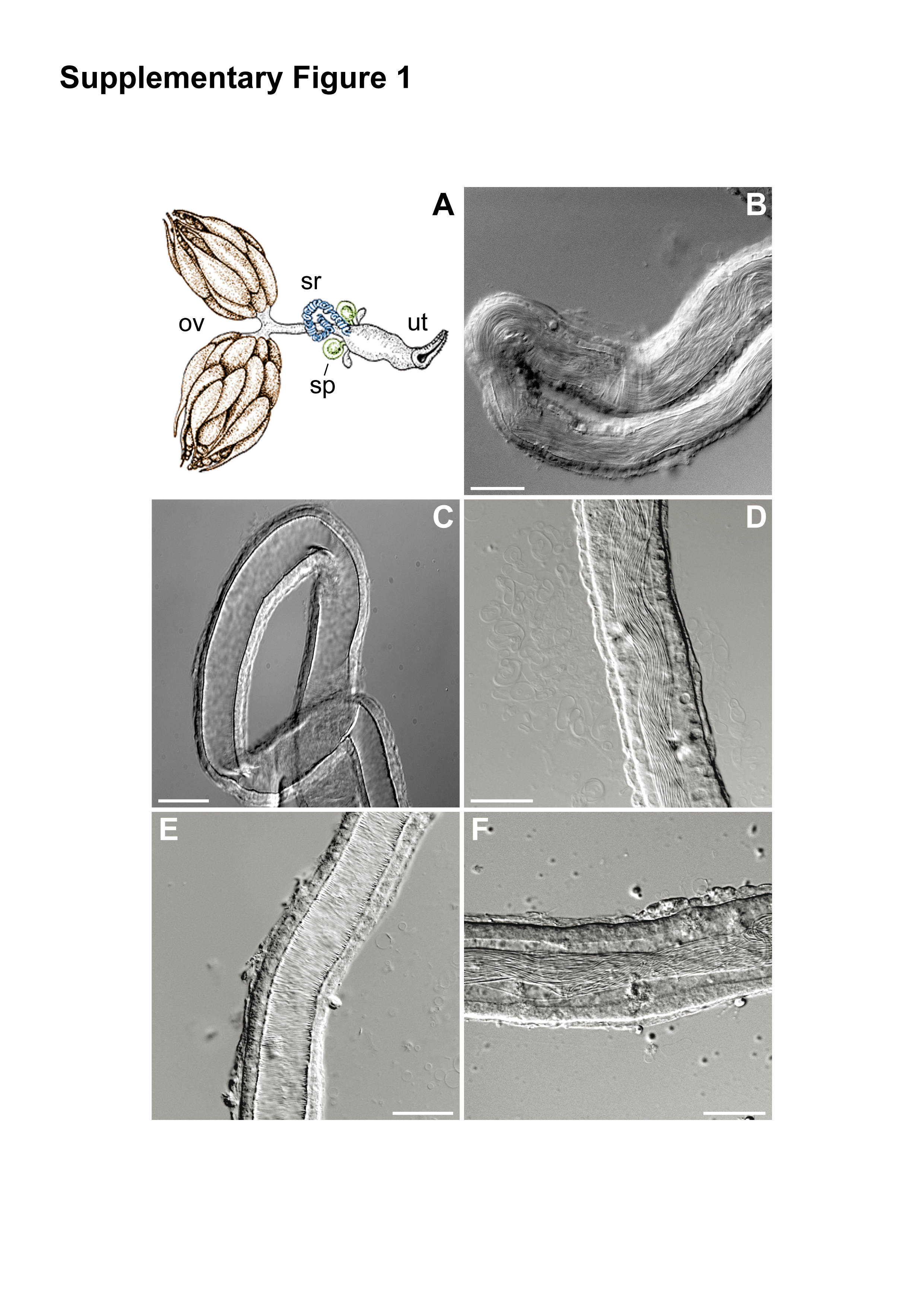

Supplement: Figure S1 — Sperm Storage Organs Dissected From Females Mated With Wild Type or amo−/− Flies. A. Schematic model of the female reproductive system. Ovary (ov), spermathecae (sp), seminal receptacle (sr), uterus (ut). B–F. Seminal receptacles dissected 30–60 minutes after observed mating. Wild type virgin females were mated to males of different genotypes as indicated: B. Wild type, C. amo −/−, D. amo −/− ;P[amo], E. amo −/− ;P[amoD627V], and F. amo −/− ;P[amo/amoD627V]. (TIF) [file pone.0020031.s001.tif]

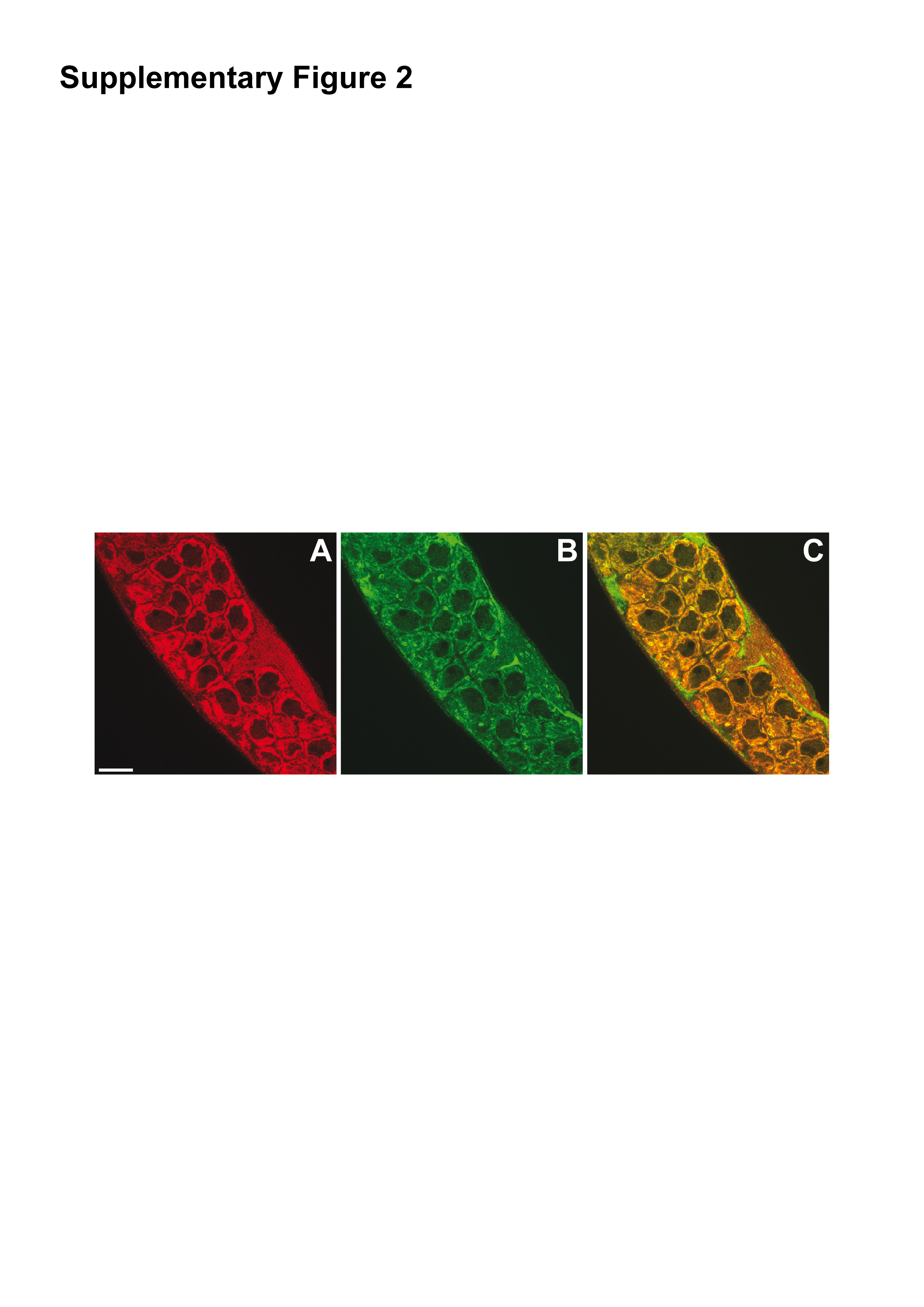

Supplement: Figure S2 — Amo Localizes to the Endoplasmic Reticulum (ER) in Spermatocytes. A. Intracellular localization of Amo in spermatocytes (Anti-Amo 1∶3000, scale bar 20 µm). B. Expression pattern of the ER marker PDI-GFP in spermatocytes. C. Merged images. (TIF) [file pone.0020031.s002.tif]

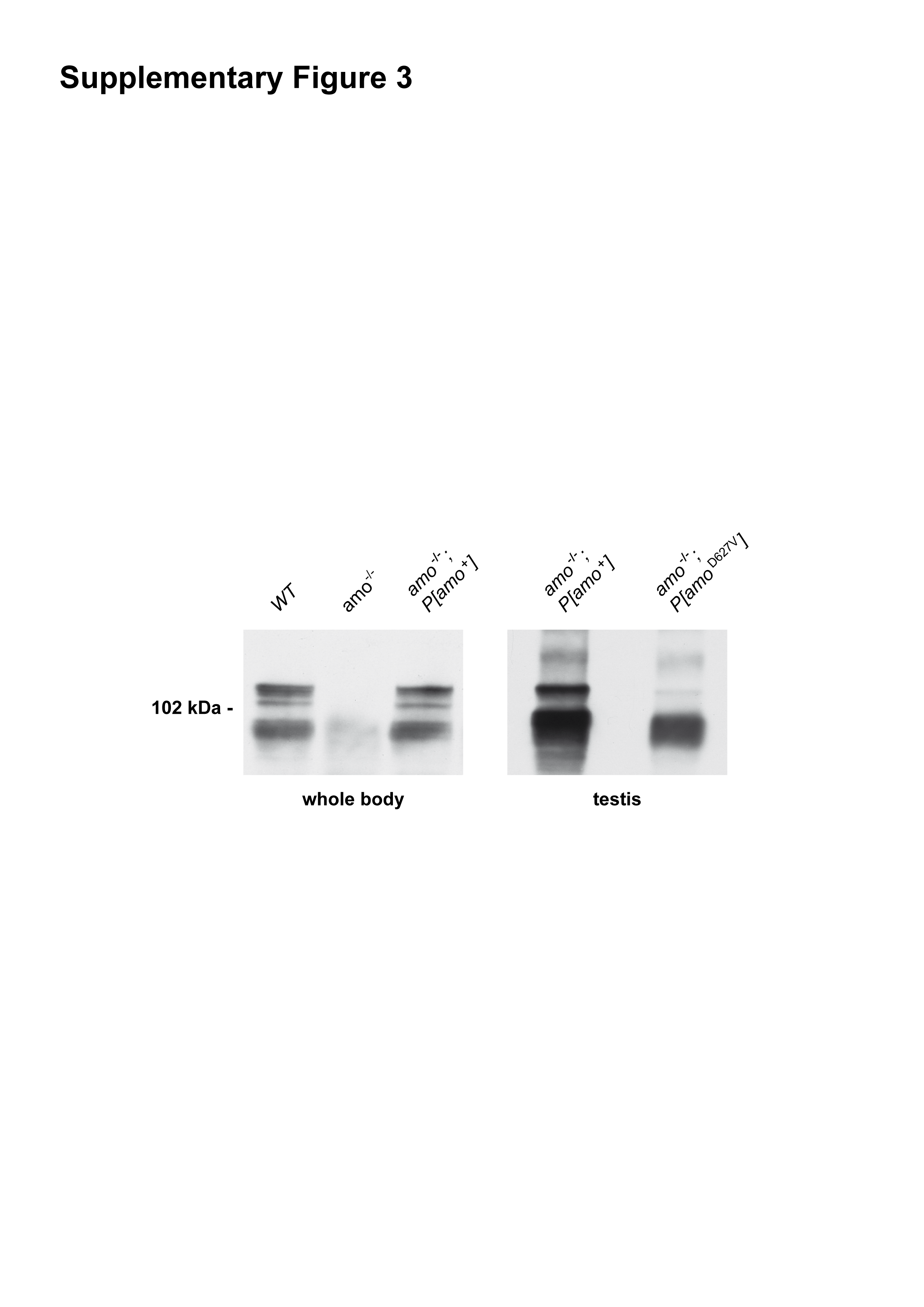

Supplement: Figure S3 — Amo Expression by Western Blot Analysis. Lysates were prepared from male flies of various genotypes and subjected to immunoprecipitation with anti-Amo antisera. Western blots were probed with anti-Amo antisera. (TIF) [file pone.0020031.s003.tif]

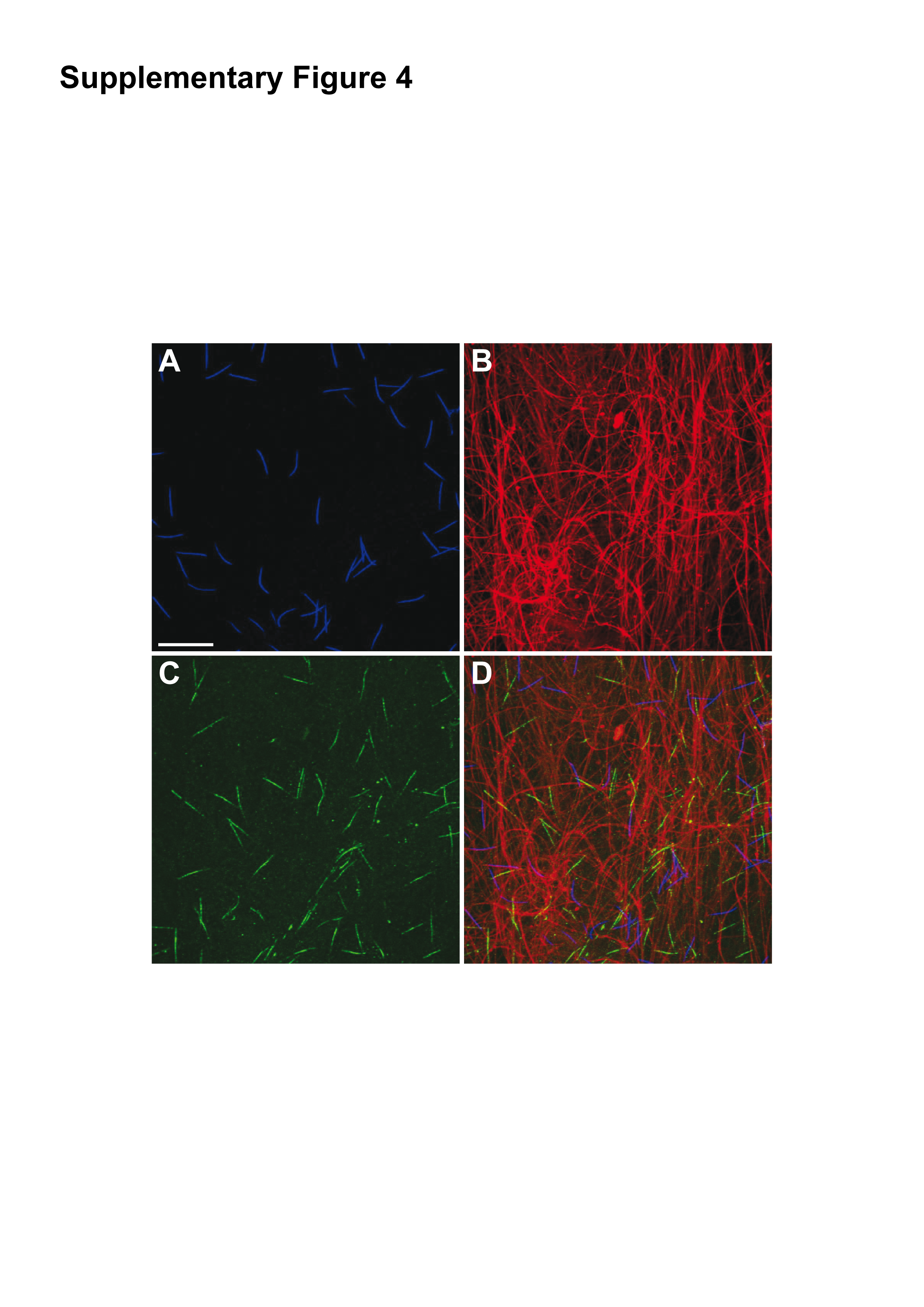

Supplement: Figure S4 — Subcellular Localization of Amo in Amo/AmoD627V Transheterozygous Sperm. Immunofluorescent labeling of sperm of the genotype amo −/− ; P[amo/amoD627V]. A. DAPI. B. Concavalin A. C. Anti-Amo. D. Merged image. Scale bar 20 µm. (TIF) [file pone.0020031.s004.tif]

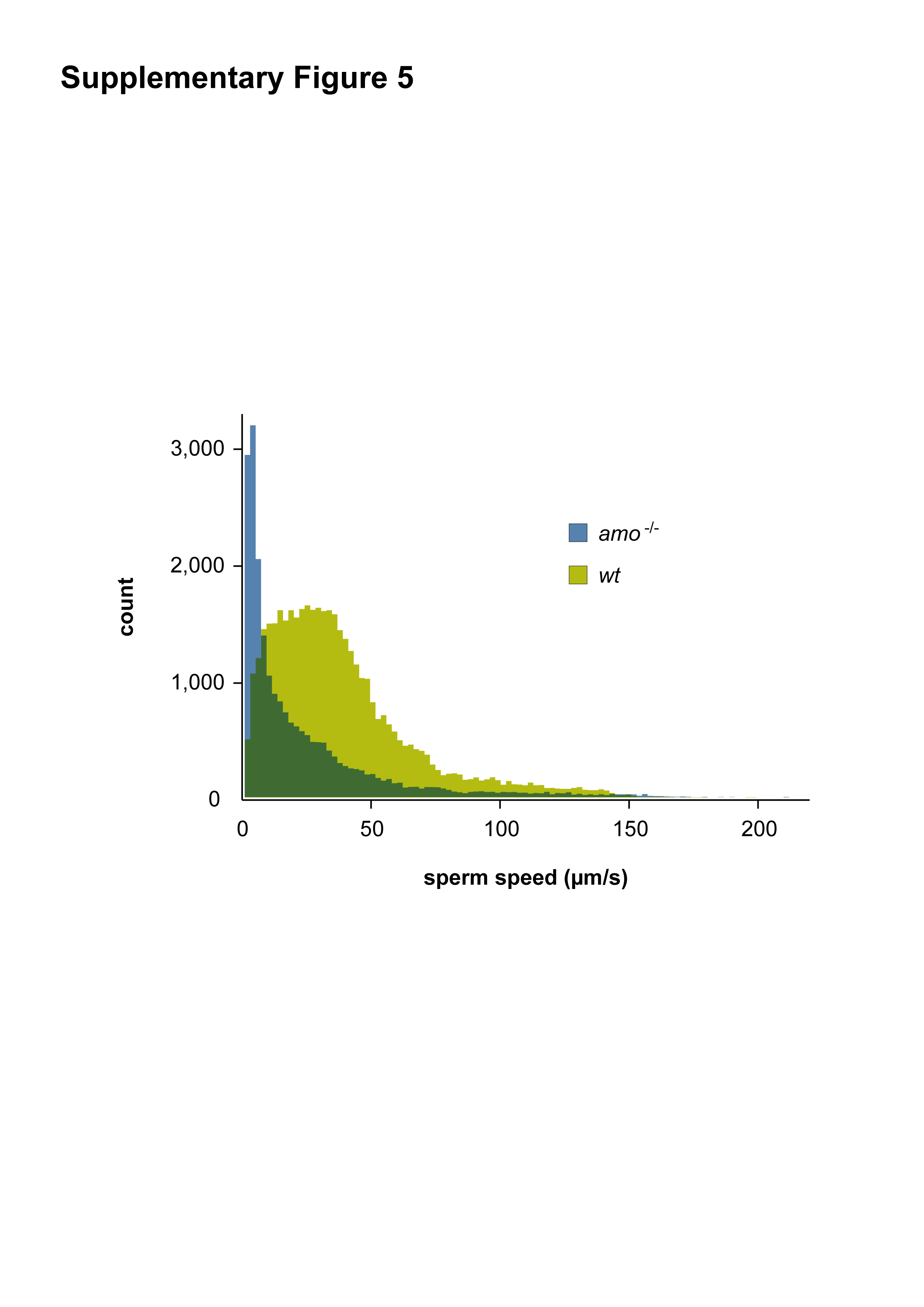

Supplement: Figure S5 — Analysis of Sperm Speed in the Uterus. Frequency distribution of sperm speed in the female reproductive tract (wt: green, amo −/−: blue, N = 7 for each genotype). (TIF) [file pone.0020031.s005.tif]

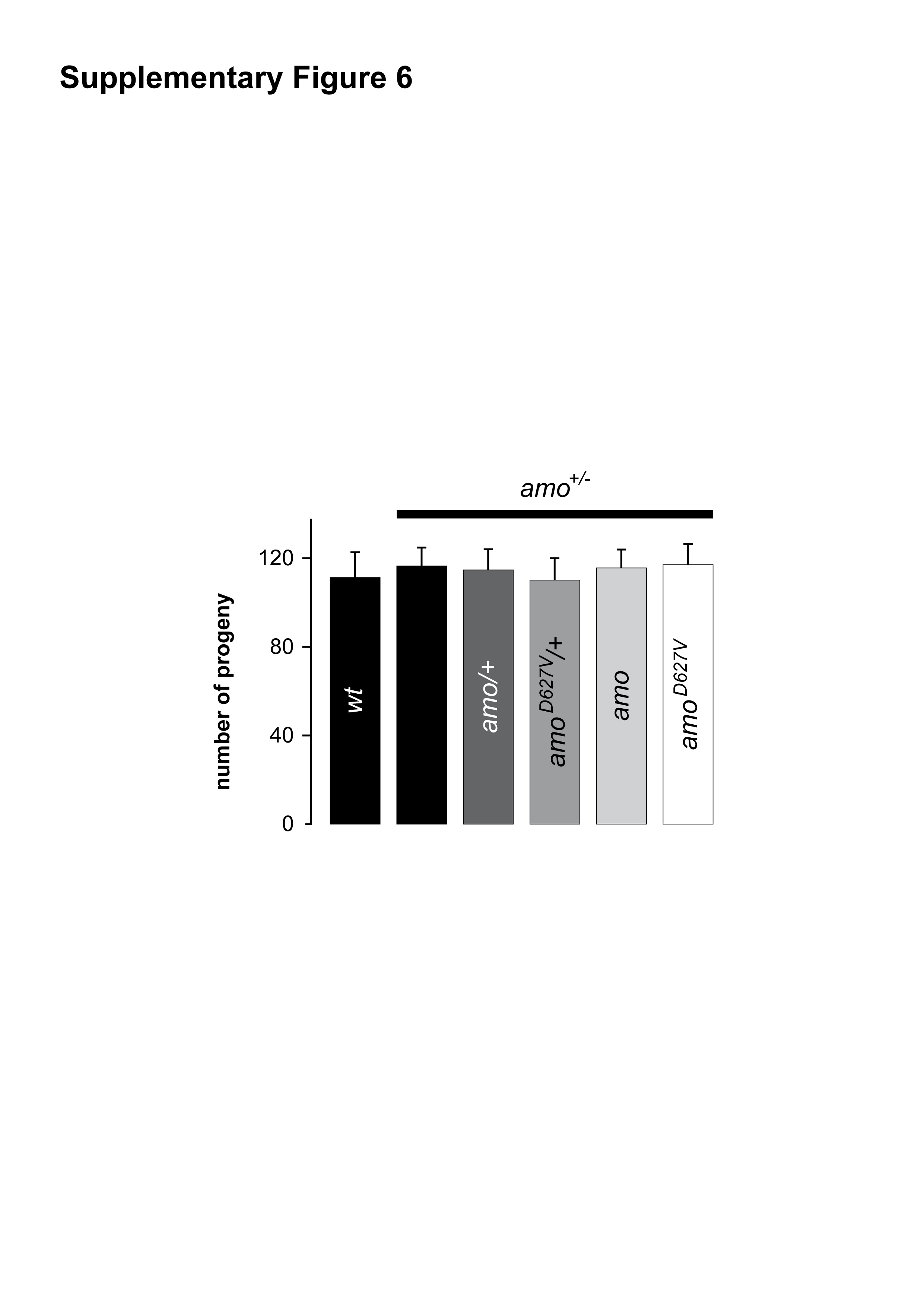

Supplement: Figure S6 — Absence of a Dominant Negative Effect of AmoD627V. Fertility tests using heterozygous amo+/ − mutant males show that introduction of one (dark grey bars) or two copies (light grey bars) of transgenic AmoD627V (3rd chromosome) does not result in impaired fertility. (TIF) [file pone.0020031.s006.tif]
